# A Novel Microbial Zearalenone Transformation through Phosphorylation

**DOI:** 10.3390/toxins13050294

**Published:** 2021-04-21

**Authors:** Yan Zhu, Pascal Drouin, Dion Lepp, Xiu-Zhen Li, Honghui Zhu, Mathieu Castex, Ting Zhou

**Affiliations:** 1 Guelph Research and Development Centre, Agriculture and Agri-Food Canada, Guelph, ON N1G 5C9, Canada; yan.zhu@canada.ca (Y.Z.); dion.lepp@canada.ca (D.L.); xiu-zhen.li@canada.ca (X.-Z.L.); honghui.zhu@canada.ca (H.Z.); 2 Lallemand Inc., Montréal, QC H1W 2N8, Canada; pdrouin@lallemand.com (P.D.); mcastex@lallemand.com (M.C.)

**Keywords:** zearalenone, microbial transformation, phosphorylation, silage, *Bacillus*

## Abstract

Zearalenone (ZEA) is a mycotoxin widely occurring in many agricultural commodities. In this study, a purified bacterial isolate, *Bacillus* sp. S62-W, obtained from one of 104 corn silage samples from various silos located in the United States, exhibited activity to transform the mycotoxin ZEA. A novel microbial transformation product, ZEA-14-phosphate, was detected, purified, and identified by HPLC, LC-MS, and NMR analyses. The isolate has been identified as belonging to the genus *Bacillus* according to phylogenetic analysis of the 16S rRNA gene and whole genome alignments. The isolate showed high efficacy in transforming ZEA to ZEA-14-phosphate (100% transformation within 24 h) and possessed advantages of acid tolerance (work at pH = 4.0), working under a broad range of temperatures (22–42 °C), and a capability of transforming ZEA at high concentrations (up to 200 µg/mL). In addition, 23 *Bacillus* strains of various species were tested for their ZEA phosphorylation activity. Thirteen of the *Bacillus* strains showed phosphorylation functionality at an efficacy of between 20.3% and 99.4% after 24 h incubation, suggesting the metabolism pathway is widely conserved in *Bacillus* spp. This study established a new transformation system for potential application of controlling ZEA although the metabolism and toxicity of ZEA-14-phosphate requires further investigation.

## 1. Introduction

Zearalenone (ZEA), chemically characterized as a macrocyclic β-resorcyclic acid lactone, is an estrogenic mycotoxin produced by *Fusarium* spp. [1]. ZEA contamination occurs in maize and other crops such as barley, oat, wheat, sorghum, millet and rice [2]. Silage, moldy hay and grain used as concentrates in animal feeds may also contain the toxin carried over from contaminated crops [3,4]. According to the European Food Safety Authority (EFSA) [5], ZEA was detected in 15% of 9877 unprocessed grains and 13,075 of food samples collected in 19 European countries between 2005 and 2010. In that survey the average ZEA level detected in unprocessed corn was 87 µg/kg with the maximal concentration as high as 2700 µg/kg. Ensiling is a common agricultural practice for the preservation of green fodders intend for livestock feed by fermentation. Although ZEA-producing fungi are inhibited by low pH and anaerobic conditions, contamination by ZEA is not mitigated during the process [6]. Moreover, these conditions (i.e., acidic and anaerobic environment) bring challenges for ZEA detoxification by microorganisms.

Strategies for ZEA decontamination have been investigated for several decades and can be categorized into chemical, physical, and biological approaches. Among them, efficient, specific, and environmentally friendly biological detoxification methods, which transform ZEA into less toxic metabolites, are being actively investigated [7,8]. Biological transformation of ZEA was reported in a variety of microorganisms such as bacteria, yeasts, or filamentous fungi (molds). These organisms included strains of *Bacillus* [9,10,11,12,13], *Pseudomonas* [14,15], *Acinetobacter* [16], *Rhodococcus* [17], *Saccharomyces* [18], *Aspergillus* [19,20,21], *Rhizopus* [19,22], *Trichosporon* [23], *Clonostachys* [24], and *Thamnidium* [25]. Among the identified metabolites, α-zearalenone (α-ZOL), β-zearalenone (β- ZOL), α-zearalanone (α-ZAL), and β-zearalanone (β- ZAL) have been shown to possess similar or higher estrogenic activity than ZEA. Only two conjugate metabolites (zearalenone-14-O-β-glucoside, and zearalenone-sulfate) [20,25] and two macrocyclic ring cleavage products [23,24] were demonstrated to have lower or no estrogenic effects using in vitro approaches.

The present work reported the discovery of a novel ZEA transformation pathway, ZEA phosphorylation, and the isolation, identification and characterization of a new strain of *Bacillus* sp. with ZEA phosphorylation functionality. In addition, the research demonstrated that multiple *Bacillus* species/strains also possess such functionality, opening the door for future applications of this newly discovered ZEA transformation mechanism.

## 2. Results

### 2.1. Isolation of ZEA Transforming Bacteria

ZEA reduction (>50%) was detected in seven of the 104 corn silage samples tested. One sample, labelled S62, showed 100% ZEA reduction. When tested in nutrient broth with mineral salts (NBM) at 28 °C for 5 days, the microbial consortium prepared from S62 transformed ZEA completely to a product identified below, even after being diluted 10^4^-fold. The active microbial consortium was then used for isolating single colonies by dilution plating on NBM plates. One of the selected single colonies, named S62-W, showed 100% ZEA transformation activity after 5 d incubation at 28 °C. When tested under the same temperature conditions with 120 RPM shaking, S62-W completely transformed ZEA within only 24 h.

### 2.2. Taxonomic Identity of the Active Isolate S62-W

**Figure 1:**
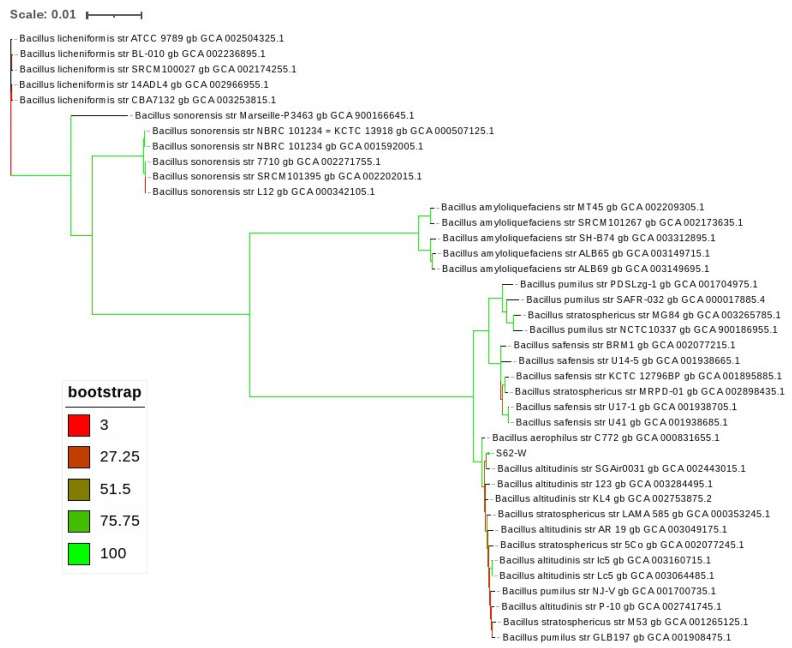
Phylogenetic analysis based on core genome sequence alignments of S62-W and 39 *Bacillus* reference genomes.

Phylogenetic analysis based on the 16S rRNA gene sequence putatively identified the active isolate S62-W to the genus *Bacillus*, with 99% identity to sequences from the *Bacillus pumilis* group [26], including *B. aerius*, *B. stratosphericus*, *B. altitudinis*, *B. pumilus*, and *B. safensis* (Table 1). Further identification of S62-W using whole genome sequencing was initially performed with the JSpeciesWS Tetra correlation search (TCS) tool, which compares a query genome against a database of reference genomes based on tetranucleotide frequencies. This method identified 19 reference genomes with a Z-score higher than 0.999, which were all identified as *Bacillus* spp., including *B. stratosphericus, B. pumilus, B. altitudinis* and *B. cellulasensis*. The average nucleotide identities between S62-W and the top fourteen TCS hits, based on BLAST alignments are given in Table 2. Phylogenetic analysis based on core genome sequence alignments of S62-W and 39 *Bacillus* reference genomes placed S62-W in a separate cluster from *B. pumilus* and *B. safensis*, in contrast to the 16S rRNA gene-based results, and within a cluster consisting mainly of *B. stratosphericus* and *B. altitudinis* strains, indicating that it is more closely related to these two species (Figure 1).

### 2.3. Identification of ZEA Transformation Product

**Figure 2:**
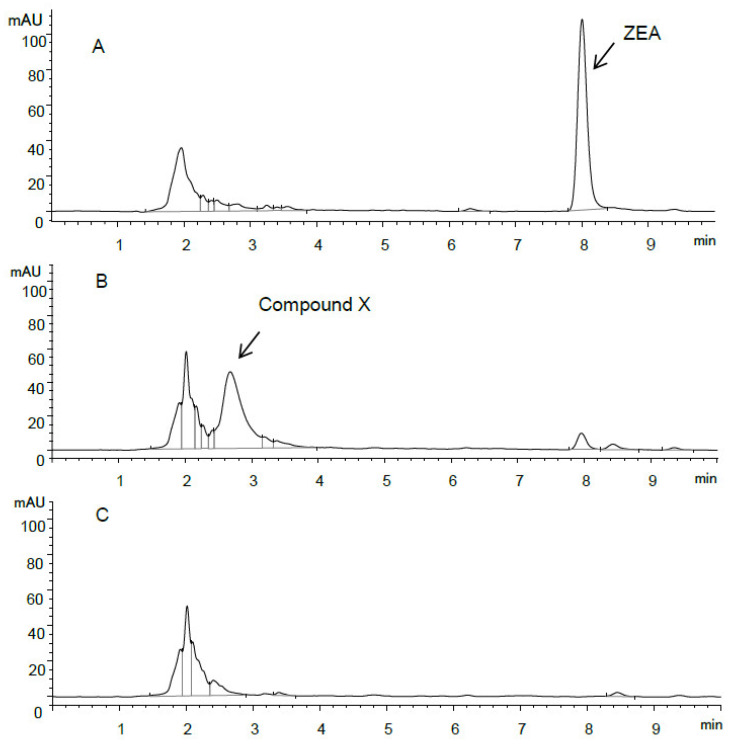
HPLC chromatogram of (**A**) S62-W culture spiked with 50 µg/mL of ZEA (0 h), (**B**) S62-W culture spiked with 50 µg/mL of ZEA (24 h), and (**C**) S62-W culture without ZEA (24 h).

HPLC chromatograms of ZEA and ZEA transformation products are shown in Figure 2. Compared to the initial culture spiked with 50 µg/mL of ZEA (Figure 2A), the presence of an unknown peak was reported together with the decrease of the peak corresponding to ZEA after 24 h incubation (Figure 2B). Given that the peak was not observed in the control sample without ZEA (Figure 2C), this indicated that the corresponding ZEA transformation product, compound X, was related to the metabolism of ZEA, resulting from ZEA transformation by the isolate S62-W. The earlier retention time suggested that compound X was characterized as having a higher polarity than ZEA.

**Figure 3:**
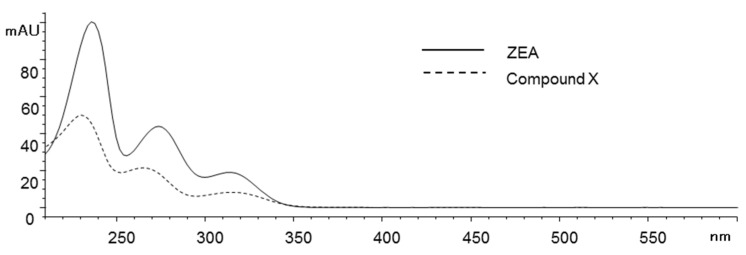
HPLC UV spectra of ZEA and compound X.

In the UV spectra of ZEA and compound X (Figure 3), both compounds showed similar triple absorption peaks in the UV wavelength range, although the hypsochromic shift of compound X was observed. This observation suggested that compound X shares a similar structure to ZEA.

**Figure 4:**
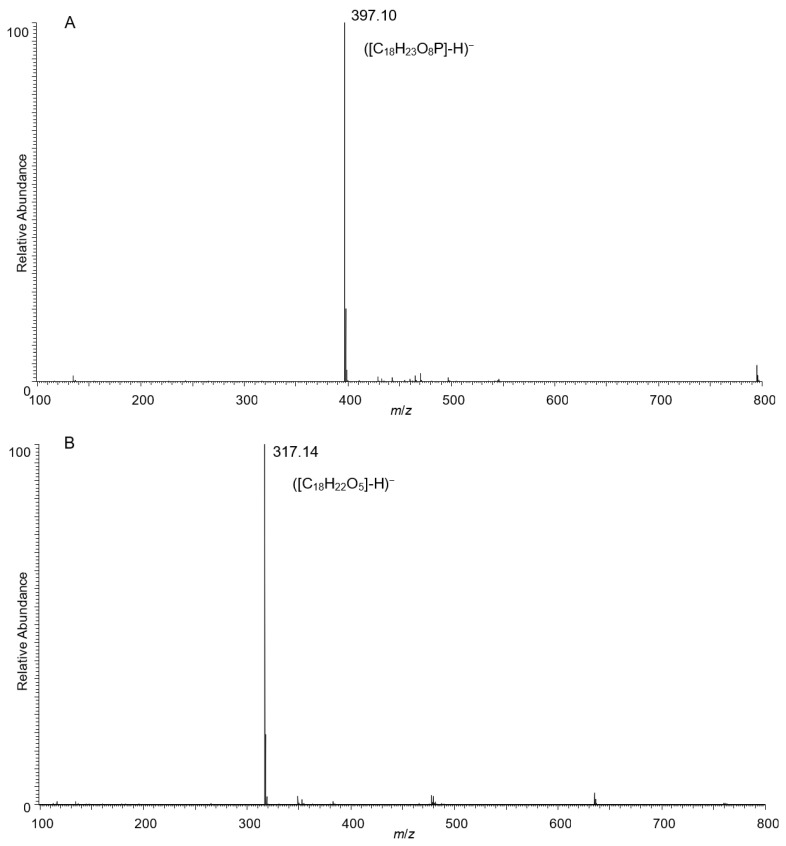

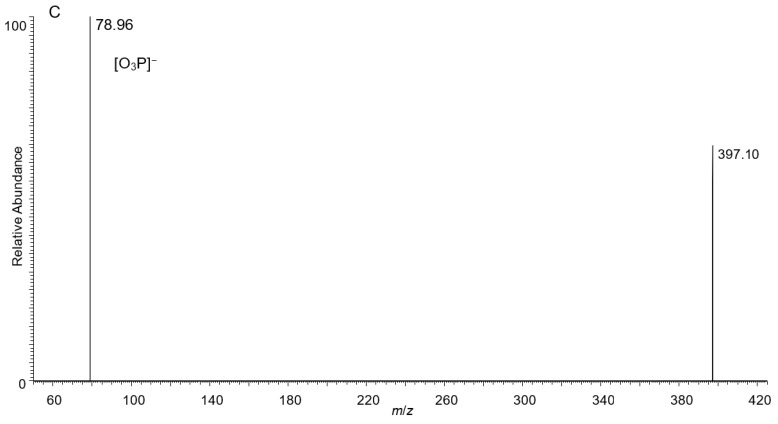
MS spectra of (**A**) standard ZEA (25 µg/mL) and (**B**) compound X (25 µg/mL); MS/MS spectrum of (**C**) compound X.

The LC-MS/MS analyses of the standard ZEA and purified compound X are presented in Figure 4. Compared to the major *m*/*z* signal of ZEA (317.14), a higher *m*/*z* signal of 397.10 was observed in the MS spectrum of compound X, which indicated that it is probably a ZEA conjugate. The major *m*/*z* signal of 78.96 in the MS/MS spectrum of fragment ions originally from the precursor ion of ([C_18_H_23_O_8_P]-H)^−^ revealed a substitution of a phosphate group of ZEA.

The purified compound X was analyzed by nuclear magnetic resonance (NMR). The 1D^1^H and 1D^13^C NMR spectra, along with correlations in the associated 2D NMR spectra, were used to assign resonances to all non-exchangeable ^1^H and ^13^C nuclei. The 2D correlations in the associated COSY, HSQC, HMBC and ROESY spectra were used to determine the structure of compound X. The results determined that the main structure of compound X (except for the phosphate group) is identical to that of ZEA (Table 3). A detail of particular importance was a ROESY correlation between H11 and H13 and an HMBC correlation between H12 and C13. These were used to distinguish the olefin substitution site at the phenyl ring of compound X and to confirm the assignment of the H13 and H15 phenyl ring protons at 6.75 ppm and 6.65 ppm, respectively. HMBC correlations between both H13 and H15 to the ^13^C resonance at 157.33 ppm were used to positively identify this resonance as arising from the C14, the oxygen-substituted phenyl ring carbon that is para to the ester substitution site on the phenyl ring of compound X. The ^3^J_HH_ COSY correlations, along with HMBC correlations between H6 to C8 and C7, H8 to C7, and H19 to C4, helped to differentiate the methylene ^1^H and ^13^C resonances in the aliphatic portions of compound X and to distinguish the C7 ketone ^13^C resonance (216.18 ppm). Further HMBC correlations between H13 and a ^13^C resonance at 131.54 ppm helped to establish this ^13^C resonance as belonging to the olefinic C12 carbon directly bound to the phenyl ring of compound X. The ester carbonyl resonance at 171.27 ppm was identified via HMBC correlations to H3 (5.01 ppm), H15 (6.65 ppm), and H13 (6.75 ppm). In general, HSQC ^1^J_CH_ crosspeaks were used to assign the remaining ^13^C resonances. The 1D^31^P NMR spectrum revealed a resonance of phosphate at ^31^P nucleus although the phosphate substitution site on the phenyl ring of compound X was not identified. The phosphate substitution site could be at either C14 or C16.

**Figure 5:**
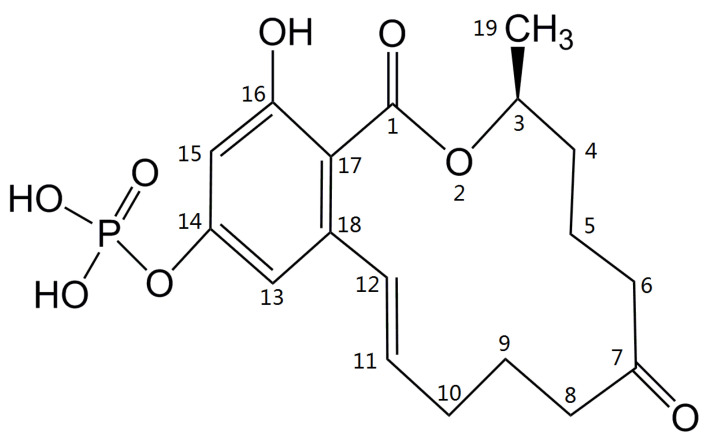
Structure of ZEA-14-phosphate.

The retention time of compound X, synthesized ZEA-14-phosphate, and ZEA-16-phosphate in HPLC chromatograms were 17.8, 17.8, and 10.1 min, respectively, which indicated that the structure of compound X and ZEA-14-phosphate is probably identical. With the combination of HPLC, LC-MS/MS and NMR analyses, the compound X is confidently identified as the phosphate conjugate at C14 position of ZEA (Figure 5), which is named ZEA-14-phosphate (ZEA-14-P).

### 2.4. ZEA Transformation by Bacillus sp. S62-W in Media

**Figure 6:**
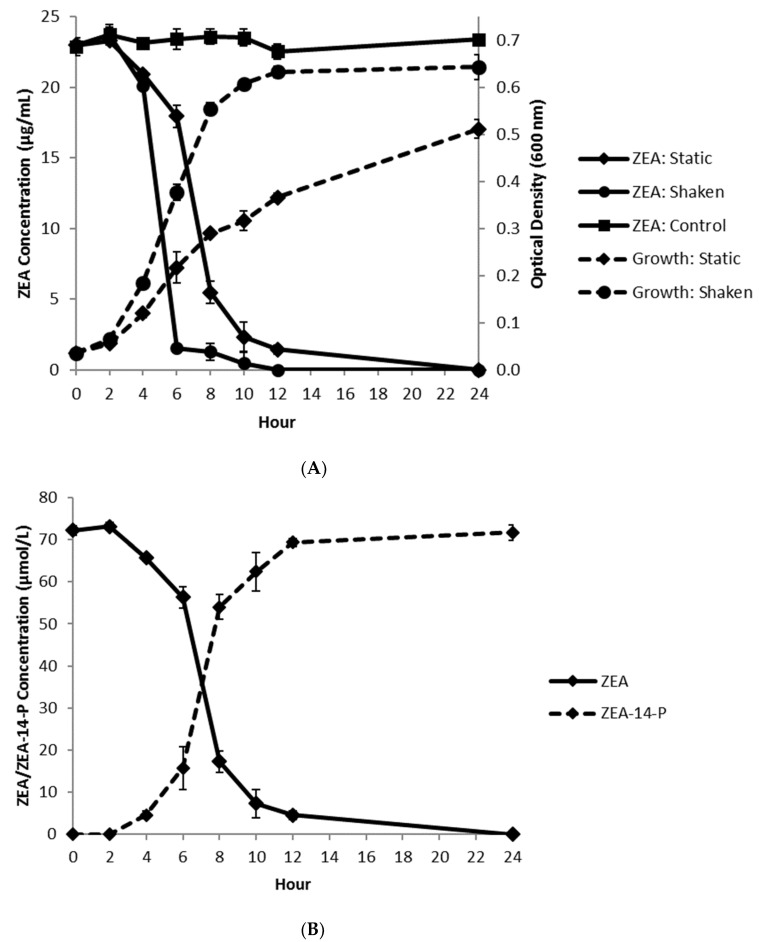
Correlation (**A**) between ZEA decrease and growth of *Bacillus* sp. S62-W in CMB at 28 °C (*n* = 3), and transformation (**B**) of ZEA and production of ZEA-14-phosphate by *Bacillus* sp. S62-W in CMB at 28 °C (*n* = 3).

When tested with the initial concentration of ZEA and bacterial cells at 25 µg/mL (78.5 µmol/L) and 2.5 × 10^7^ cfu/mL, respectively, the decrease of ZEA was found to be positively correlated with the optical density value of *Bacillus* sp. S62-W cells in the culture in both static and shaken incubations (Figure 6A). This observation indicated that the transformation was mainly related to the primary metabolism of the bacterial isolate, indicating that the cell growth of *Bacillus* sp. S62-W could facilitate ZEA transformation.

The transformation product, ZEA-14-phosphate (i.e., compound X), was detected and quantified by HPLC. A negative correlation between the concentrations of ZEA and ZEA-14-phosphate was observed (Figure 6B). Since no intermediate compounds were detected, this suggests that ZEA was fully transformed into ZEA-14-phosphate by *Bacillus* sp. S62-W. The efficiency of this transformation was high since 100% of ZEA was transformed within 24 h.

### 2.5. Effects of Environmental Factors on ZEA Transformation

**Figure 7:**
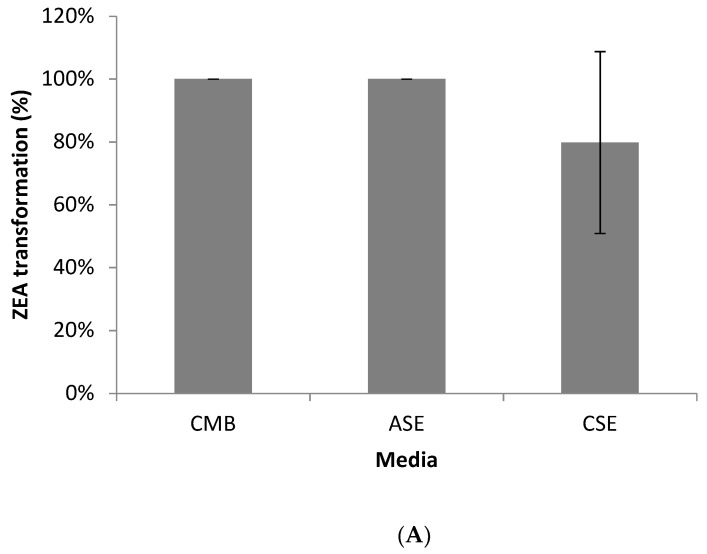

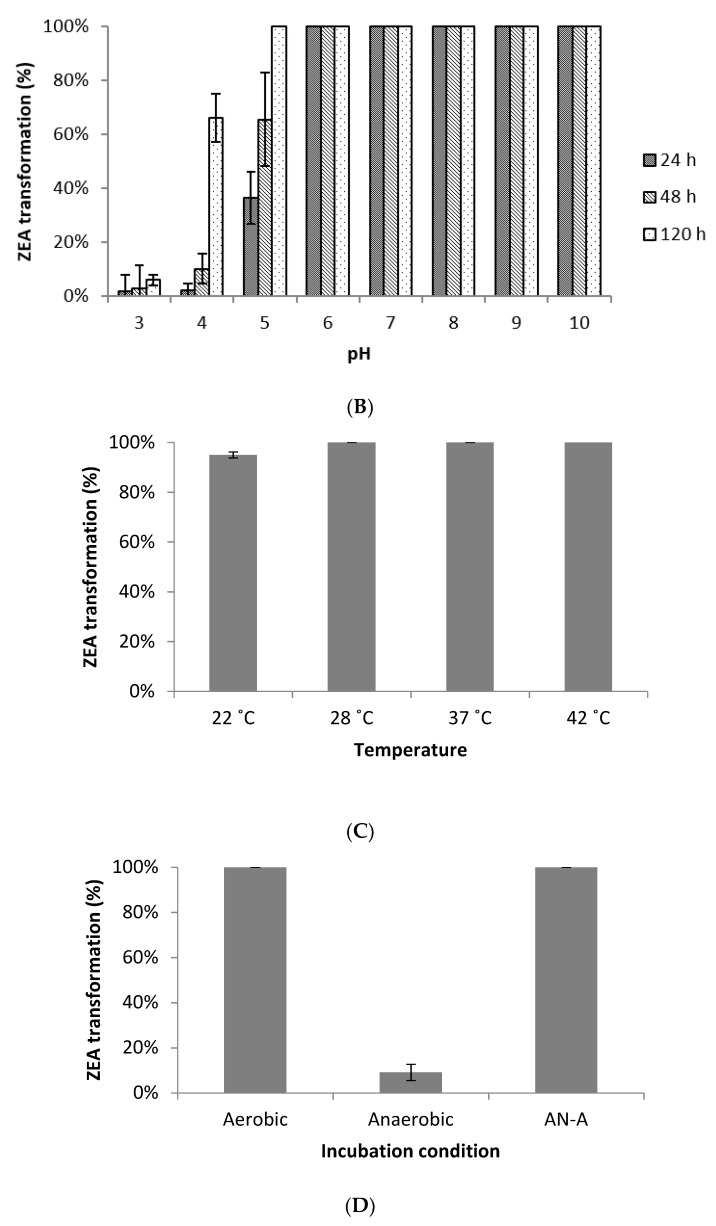

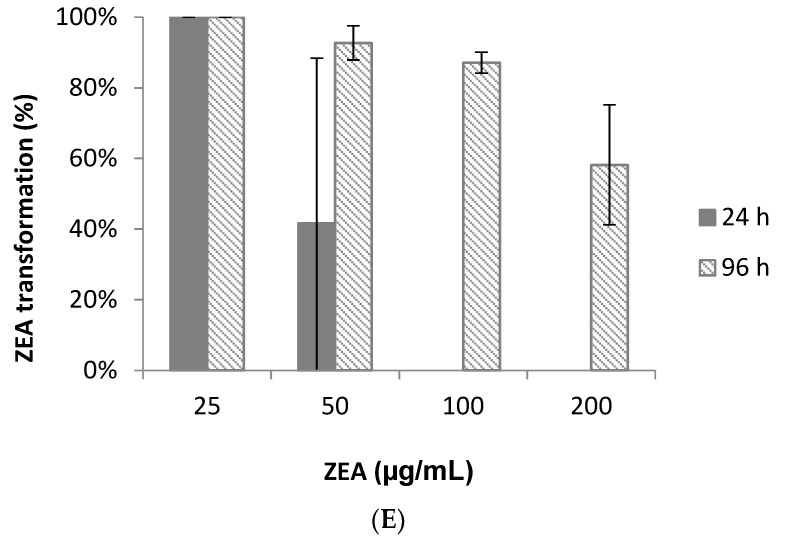
(**A**): ZEA transformation by *Bacillus* sp. S62-W in various media (28 °C, 3 days, *n* = 3). CMB: Corn meal broth W/O yeast extract; ASE: Alfalfa silage extract; CSE: Corn silage extract. (**B**): ZEA transformation by *Bacillus* sp. S62-W in CMB with pH value between 3 and 10 (28 °C, 5 days, *n* = 3). (**C**): ZEA transformation by *Bacillus* sp. S62-W in CMB incubated between 22 and 42 °C (2 days, *n* = 3). (**D**): ZEA transformation by *Bacillus* sp. S62-W in CMB under different incubation conditions (*n* = 3). Aerobic: Aerobic incubation at 28 °C for 2 days; Anaerobic: Anaerobic incubation at 28 °C for 2 days; AN-A: Anaerobic incubation at 28 °C for 6 days following by aerobic incubation at 28 °C for 2 days. (**E**): ZEA transformation by *Bacillus* sp. S62-W in CMB spiked with concentration varying between 25 and 200 µg/mL of ZEA (28 °C, 24 h and 96 h, *n* = 3).

Environmental factors, including culture media, pH, temperature, oxygen requirement and initial mycotoxin concentration, had different effects on the ZEA transformation by *Bacillus* sp. S62-W. Figure 7A shows the level of ZEA transformation in different media. Full transformation activities were observed in corn meal broth (CMB) and alfalfa silage extract (ASE). Corn silage extract (CSE) supported a lower transformation rate (80%), likely due to its higher concentration of organic acids and lower level of nutrients.

The pH values affected the ZEA transformation capacity (Figure 7B). Complete transformation was achieved within 24 h at 28 °C under pH values ranging between 6.0 and 10.0. At a lower pH of 4.0, however, noteworthy transformation appeared only after 120 h of incubation. The results revealed that the acid tolerance of *Bacillus* sp. S62-W was greatly reduced at pH below 5.0 and the ZEA transformation activity by the bacterial isolate was low in acid medium.

Since temperature in silage may reach as high as 40 °C upon oxygen ingress, the capability of *Bacillus* sp. S62-W to transform ZEA was evaluated at a broad range of temperatures between 22 °C and 42 °C (Figure 7C). Under all temperatures tested, more that 95% of ZEA was transformed to ZEA-14-phosphate after 24 h static incubation, indicating that the bacterial isolate can still be active under relatively high temperatures.

The oxygen requirement for ZEA transforming activity was evaluated (Figure 7D) as the ensiling process, comprising both aerobic (aerobic phase and feed-out phase) and anaerobic stages. The full transformation activity was reached under aerobic incubation. On the contrary, less than 10% ZEA was transformed under anaerobic conditions. However, transformation activity can be restored by aerobic incubation. Complete transformation was achieved after incubating the bacterial strain under anaerobic condition for 6 d following by a 2 d aerobic incubation. Although the isolate may not function during the absolute anaerobic stage, it may potentially exhibit transformation activity in the early stages of ensiling immediately after inoculation of the harvested forage, as well as during short exposure to air once the silage is open at feed-out.

Although the ZEA contamination levels in crops rarely exceed 5 mg/kg, the contaminated areas invaded by mycotoxin-producing fungi may accumulate higher concentrations of ZEA. Thus, the maximum capacity of ZEA transformation of S62-W was investigated. After 96 h aerobic incubation in NBM, more than 80% of ZEA transformation was observed in the samples spiked with up to 100 µg/mL of ZEA. In the sample with 200 µg/mL of ZEA, the transformation ratio was still greater than 50% (Figure 7E).

### 2.6. Determination of ZEA Phosphorylation in Various Bacillus Strains

**Figure 8:**
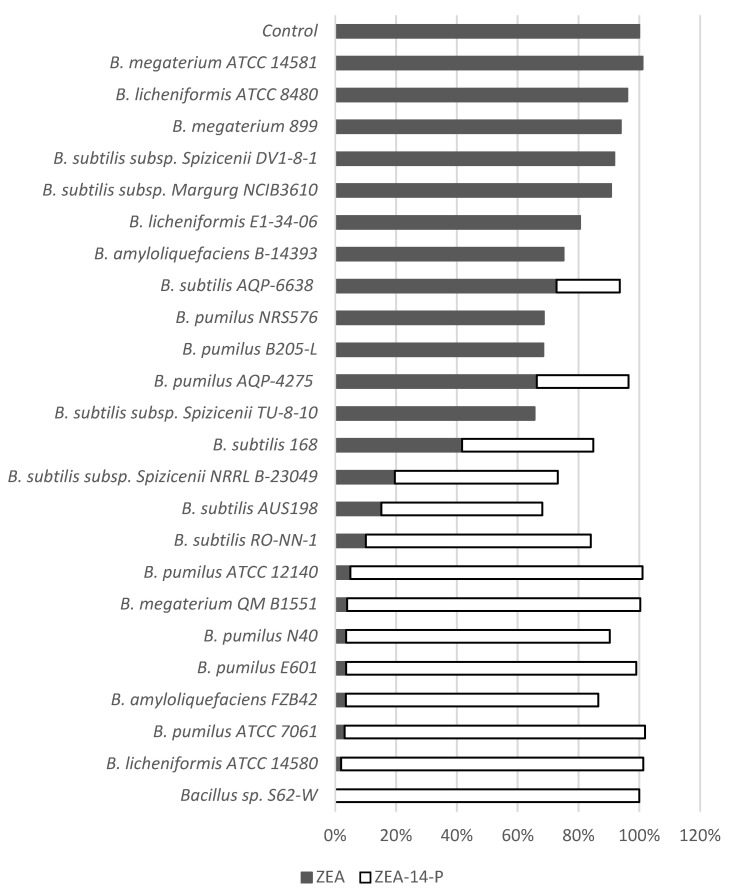
ZEA phosphorylation activity of 23 *Bacillus* spp. and *Bacillus* sp. S62-W in CMB at 28 °C for 24 h incubation.

Among the 23 strains of *Bacillus* tested, 13 exhibited phosphorylation activity at an efficacy between 20.3% and 99.4% after 24 h incubation at 28 °C, suggesting that the metabolism pathway is widely conserved among *Bacillus* spp. Five *Bacillus* spp. including *B. licheniformis* ATCC 14580, *B. pumilus* ATCC 7061, *B. megaterium* QM B1551, *B. pumilus* ATCC 12140, and *B. pumilus* E601 possess high phenotypic capability (>95%) to transform ZEA to ZEA-14-phosphate (Figure 8). Two commercial strains, *B. pumilus* AQP-4275 and *B. subtilis* AQP-6638 displayed partial transformation of 30.2% and 20.8%, respectively. *B. subtilis* subsp. Spizicenii NRRL B-23049 and *B. subtilis* AUS198, reduced 80.4% and 84.8% of spiked ZEA, but only 53.6% and 52.9% of ZEA-14-phospate were detected after incubation. The notable difference (more than 20%) between the decrease of ZEA and production of ZEA-14-phosphate indicated unidentified intermediate or final products in the culture.

## 3. Discussion

The disappearance of ZEA through the metabolism of *Bacillus* spp. has been reported in many studies [9,10,11,12,13]. However, the lack of a degradation mechanism limited further investigations and application since the identification and safety assessments of the transformation products are essential to develop an effective biological detoxification system [27]. In our study, the phosphorylation of ZEA revealed a novel transformation mechanism through *Bacillus* spp. Phosphorylation is critical for many cellular processes through the addition of a phosphoryl group to proteins, sugars, lipids, and other molecules, during many metabolic and cell signaling pathways [28,29]. However, the metabolism of xenobiotic compounds through phosphorylation has rarely been reported compared to other conjugations [28]. Two isoflavones, daidzein and genistein, were found to be transformed to daidzein-7-O-phosphate and genistein-7-O-phosphate through attachment of a phosphate moiety to the phenolic hydroxyl group by *B. subtilis* [30], which represents some of the limited examples of phosphate conjugates produced by bacteria. The well-known phase II reactions of xenobiotic metabolism in mammals include glucuronidation, glycosidation, sulfation, methylation, acetylation, amino acid conjugation, glutathione conjugation, and fatty acid conjugation [31]. Phosphorylation is not typically included in these reactions, although rare cases (e.g., phosphorylation of chloramphenicol and 2-acetamidofluorene) have been reported [28]. Conjugated products of ZEA through microbial metabolic processes are limited to ZEA-14-O-β-glucoside and ZEA-sulfate, which were transformed by *Rhizopus* sp. and *Aspergillus niger*, respectively [20,25]. In this study, for the first time, the phosphorylated product of ZEA resulting from bacterial metabolism has been identified and purified. This distinctive and abnormal xenobiotic transformation enriches our knowledge of microbial conjugation reactions.

One issue that hinders the successful application of mycotoxin detoxification using a biological strategy is the assessment of functional microbes for safety. *Bacilli* and most lactic acid bacteria are good candidates for food-related applications, since several species are included in the EFSA Qualified Presumption of Safety list, which can be used as a source of feed additives [32]. In the current study, the bacterial strain S62-W belongs to the genus *Bacillus* and exhibits efficient ZEA transformation activity. More significantly, the main transformation product has been clearly characterized. Combined with its advantages of acid tolerance (down to a pH of 4.0), working at a range of temperatures (22-42 °C), and the capability of transforming high levels of ZEA (up to 200 µg/mL), *Bacillus* sp. S62-W may be applied to various feedstuffs including silage in the postharvest phase and during feed-out. In order to investigate the specificity of this phosphorylation mechanism in *Bacillus*, 23 *Bacillus* strains representing various species were selected and their transformation capacities tested in vitro. The widely positive results among various strains suggest that ZEA phosphorylation activity is common to phylogenetically related strains in the same sub-groups of *Bacillus,* based on 16S rDNA analysis [33]. However, several strains of the same species differed in their transformation capacity, exhibiting either low or high activity, indicating that the function is also strain specific.

*Bacillus* spp. are ubiquitous in soil and are frequently present in silage [34]. The spore-forming capacity of *Bacillus* contributes to its survival under ensiling conditions, including a prolonged period in a low pH environment as well as anaerobic conditions. The endospores germinate and the cells grow in the silage when the pH and oxygen concentration are appropriate [35,36]. Queiroz et al. [37] proposed that exposure to air results in a greater amount of *Bacillus* endospores in the outer layers of silage bales than the central and tightly-packed layers. The aerobic deterioration caused by the oxidation of lactic acid by yeasts also supports the growth of *Bacillus* spp. [37]. Although the role of *Bacillus* spp. in silage has been generally considered to be negative, they may improve the aerobic stability of silage and produce antimicrobials to inhibit fungal growth [35]. Improvements in aerobic stability were observed after inoculation of *B. subtilis* along with *Lactobacillus plantarum* [38]. In addition to the adaptation in the ensiling procedure, the *Bacillus* spp. also provided supplementary benefits as feed additives. Some *Bacillus* spp. with ZEA mitigation activities have been shown to possess probiotic characteristics, including tolerance to the gut environment and anti-pathogenic capabilities [11,39]. As an affordable source of enzymes, *Bacillus* spp. have been widely applied in enzyme production [40]. *B. licheniformis* CK1, another reported ZEA degrading microorganism, displayed high levels of xylanase, CMCase and protease activities, which improved the digestibility of nutrients in feed [9]. From a safety perspective, *Bacillus* spp. are considered ideal mycotoxin mitigation agents due to their perceived probiotic properties, although some species (e.g., *B. anthracis* and *B. cereus*) are widely known as pathogens because of the production of enterotoxins [27,41]. Since *Bacillus* sp. S62-W was originally isolated from silage, it may be adapted to silage conditions and contribute to the ensiling process and quality. Meanwhile, the variously functional *Bacillus* spp. described in the present study, particularly the two commercial *Bacillus* strains, provide multiple options for potential industrial application based on their safety profile and additional benefits.

The toxicities of conjugated ZEA transformation products have been reported in both in vitro and in vivo studies. El-Shakawy and Abul-Hajj [42] carried out an estrogen receptor affinity assay using rat uterine cells to evaluate the estrogenic activities of various ZEA derivatives. The metabolites with a blocked 14-phenolic group (14,16-dimethoxy-ZEA and ZEA-14-O-β-glucoside) were found to be biologically inactive. Similar observations were reported for less estrogenic activities of ZEA-sulfate through MCF-7 cell proliferation assays [20]. Conjugated mycotoxins are often considered “masked mycotoxins” (i.e., biological modification of mycotoxins which may release parent mycotoxins through mammalian metabolism) although the term was recently defined as biologically modified mycotoxins that were conjugated by plants [43]. If conjugation results in decreased toxicity, however, it should be designated as detoxification rather than masking [44]. For example, the mammalian phase II reactions use a strategy to detoxify xenobiotic compounds by conjugating endogenous groups, which creates more acidic and polar compounds that have lower lipid-solubility, preventing them from diffusing into cell membranes and facilitating their elimination from the body [31]. An in vitro investigation of the stability of ZEA-14-O-β-glucoside in the human gut was performed [45]. Neither hydrolysis in the digestion juice of the upper gut, nor gut epithelial absorption in Caco-2/TC7 cells, were observed. Nevertheless, the conjugated ZEA was fully hydrolyzed to ZEA by the human gut microbiota and 40%–70% of the transformed ZEA was further metabolized to unknown compounds, which revealed the potential risks of conjugated mycotoxins [45]. As a novel conjugated mycotoxin, a comprehensive toxicity assessment of ZEA-14-phosphate will need to be performed not only to understand the in vitro estrogenic activity, but also its fate following ingestion by animals.

## 4. Conclusions

The discovery of a new ZEA microbial transformation system, including the novel transformation reaction (i.e., phosphorylation) and bacterial isolate (i.e., *Bacillus* sp. S62-W) provides an alternative strategy for ZEA detoxification. The current data demonstrates that there is great potential to use such system for commercial application to mitigate the ZEA contamination problem in silage and other agricultural commodities, although further research is necessary. The toxicity reduction of ZEA-14-phosphate needs to be demonstrated using cell culture assays and animal trials. Strategies to apply the transformation system under commercial conditions need to be developed in future studies.

## 5. Materials and Methods

### 5.1. Chemicals and Culture Media

ZEA was obtained from Sigma-Aldrich (St. Louis, MO, USA). ZEA-14-phosphate and ZEA-16-phosphate were chemically synthesized by TripleBond (Guelph, ON, Canada) according to the proposed ZEA transformation products in this study. NBM contained 8 g nutrient broth (BD, Mississauga, Canada), 2.5 g K_2_HPO_4_, 2.5 g KH_2_PO_4_, 1.0 g (NH_4_)_2_HPO_4_, 0.2 g MgSO_4_·7H_2_O, 0.01 g FeSO_4_, and 0.007 g MnSO_4_ per liter. Nutrient agar (NA) plates were prepared using 8 g of nutrient broth and 15 g agar per liter. CSE or ASE was prepared by blending 50 g corn or alfalfa silage with 200 mL of deionized water for two minutes and was filtered through a Whatman No. 1 filter paper (Whatman, Maidstone, Kent, UK). CMB was prepared by soaking 40 g of corn meal in 1 L of deionized water at 58 °C for 4 h. After standing for 2 h, the broth was filtered through a Whatman No. 1 filter paper and 3 g (NH_4_)_2_SO_4_, 1 g K_2_HPO_4_, 0.5 g MgSO_4_·7H_2_O, 0.5 g K_2_SO_4_, 0.01 g FeSO_4_, and 0.007 g MnSO_4_ were added. All the media were autoclaved at 121 °C for 15 min before using.

### 5.2. Screening of Corn Silage Samples

A total of 104 corn silage samples were collected from silos of various farms located in the states of Wisconsin, New York, Vermont, Michigan and Maine. Silage samples were screened for the ZEA transformation activity by spiking ZEA in the silage extracts. The silage extracts were prepared by weighing 50 g of each silage sample and mixing with 200 mL of sterile deionized water in a blender. The mixture was blended for 2 min and filtrated through a coarse filter paper. One hundred µL of sample was transferred into 900 µL of NBM or CSE containing 25 µg/mL of ZEA. Two incubation conditions (aerobic at 28 °C and 5% CO_2_ at 30 °C) have been applied for the screening. ZEA concentration and accumulation of ZEA transformation products were analyzed by HPLC and LC-MS methods after the incubation up to 30 days.

### 5.3. Single Colony Isolation and Purification

Samples showing decreased ZEA concentration were selected for single colony isolation. A series of 10-fold dilution was applied using saline solution (0.85% NaCl) and 100 µL of diluted culture was spread on NA plates. Plates were incubated aerobically at 28 °C for 2 days. A total of 100 colonies were randomly selected from the 10^−5^ dilution plates and transferred into NBM medium containing 25 µg/mL of ZEA. Samples were incubated aerobically at 28 °C for 5 days. The decrease of ZEA after incubation was detected by HPLC.

### 5.4. Taxonomic Identification of the Active Isolate

Genomic DNA was extracted from a single colony using InstaGene™ matrix (Bio-Rad, Mississauga, ON, Canada). The PCR primers used for 16S rRNA gene amplification were universal primers of 16S8F primer 5′ AGA GTT TGA TCC TGG CTC AG 3′ and 16S1391R primer 5′ GAC GGG CGG TGW GTR CA 3′. PCR was performed in a total volume of 25 µL, which contained 0.6 µmol/L of each primer, 1× of HotStarTaq^®^ Master Mix (QIAGEN, Toronto, ON, Canada) and 20–40 ng of DNA. Amplification was carried out in a GeneAmp^®^ PCR System 9700 (Applied Biosystems, Waltham, MA, USA) under the following conditions: 95 °C (15 min); 35 cycles of 94 °C (20 s), 52 °C (15 s), 72 °C (1 min 30 s); and a 72 °C (7 min) extension step.

Amplified PCR products with a single fragment were confirmed on a 2.0% agarose gel and purified using NucleoFast 96 PCR clean up plate (Macherey-Nagel, Düren, Germany). Purified PCR fragments were sequenced using BigDye^®^ Terminator v3.1 Cycle Sequencing method (Applied Biosystems). DNA sequencing was performed with an automated ABI 3730 DNA analyzer (Applied Biosystems) following the manufacturer’s instruction (ABI Prism^®^ DNA Sequencing Analysis Software Version 3.7, Applied Biosystems). Forward and reverse sequences were edited and aligned. Aligned sequences were compared to the NCBI database using the BLAST tool.

Genomic DNA was extracted from S62-W broth and plate cultures for high-throughput sequencing using QIAGEN^®^ Gentra Puregene Yeast/Bact. Kit. Sequencing libraries were prepared from 1 ng of genomic DNA with the Nextera XT kit (Illumina) according to the manufacturer’s instructions, and subsequently sequenced on a MiSeq instrument (Illumina, San Diego, CA, USA) using a 600-cycle v3 kit (Illumina). The resulting 300 bp paired-end reads were filtered to remove phiX, adapter and human sequence contamination using BBTools (https://jgi.doe.gov/data-and-tools/bbtools/ (accessed on 20 April 2021)) and assembled with SPAdes v. 3.11.1 [46].

Whole-genome based identification of S62-W was initially performed with the JSpeciesWS online service [47]. Further phylogenetic analysis based on core genome alignments of the S62-W sequenced genome and 39 *Bacillus* spp. reference genomes was performed with RealPhy v. 1.12 [48]. *Bacillus* spp. were selected for phylogenetic comparison based on putative taxonomic assignments from JSpeciesWS, as well as the KmerFinder tool (https://cge.cbs.dtu.dk/services/KmerFinder/) (accessed on 20 April 2021). Five representative genomes were chosen for each species, wherever available, in the event that the species in some cases had been assigned incorrectly. A maximum likelihood tree was built with RAxML v 8.1.17 [49] using rapid Bootstrap analysis based on 400 replicates, and visualized with the Interactive Tree of Life (iTOL) web service [50].

### 5.5. ZEA Transformation Activities of the Active Isolate

ZEA transformation activities of the isolate were evaluated under various media (CMB, ASE, and CSE), temperature (22, 28, 37, and 42 °C), pH (3.0, 4.0, 5.0, 6.0, 7.0, 8.0, 9.0, and 10.0), oxygen requirement (aerobic and anaerobic), and initial ZEA concentration (25, 50, 100, and 200 µg/mL). The pure culture stored in −80 °C freezer was pre-incubated in the NBM media at 28 °C overnight before carrying each transformation evaluation. The initial cell concentration in all experiments mentioned above was adjusted to 2.5 × 10^7^ cfu/mL. The percentage of ZEA transformation was calculated with the Equation (1).

(1) $$ZEA\;transformation\;\left(\%\right)=\frac{initial\;concentration-residual\;concentration\;}{initial\;concentration}\times 100$$

### 5.6. Analysis of ZEA by HPLC

Samples and standards were analyzed using an HPLC system (Agilent Technology 1200 Series, Palo Alto, CA, USA) equipped with a quaternary pump, an inline degasseµr, and a diode array detector set at 235 nm. A Phenomenex^®^ 4 µ Jupiter Proteo 90A (250 × 4.6 mm) with a C18 guard column (Torrance, CA, USA) was used for the separation. The compound of interest, ZEA in this case, was eluted using binary mobile phase set at a flow rate of 1.0 mL/min. The mobile phase was acetonitrile:water (60:40 by volume) and the injection volume was 10 μL. The retention time of ZEA was around 8.2 min.

### 5.7. Extraction and Purification of ZEA Transformation Product

The isolate with ZEA transformation activity stored in −80 °C was centrifuged at 3500× *g* for 10 min. The pellet was collected and dissolved in 1 mL of CMB. A 100 µL volume of culture was transferred into a 10 mL of CMB and pre-incubated at 28 °C with shaking at 120 RPM for 24 h. Then pre-incubated culture (10 mL) was transferred in 400 mL of CMB media spiked with 10 mg of ZEA to reach a final concentration of 25 µg/mL. The culture was aerobically incubated at 28 °C with shaking at 120 RPM for 48 h. The decrease of ZEA and increase of ZEA transformation product (i.e., compound X) were detected by HPLC.

After incubation, the culture was centrifuged at 3500× *g* for 10 min to remove the cells and particles. The supernatant was freeze-dried, and the compound X was extracted by 10 mL of 50% *v*/*v* acetonitrile. The acetonitrile extraction solution was centrifuged at 3500× *g* for 10 min to remove the precipitation. The supernatant was passed through 0.45 µm syringe filter before HPLC collection.

The filtered extraction solution was fractionally collected using an HPLC system (Agilent Technology 1200 Series, Palo Alto, CA, USA) equipped with a quaternary pump, an inline degasser, and a diode array detector set at 270 nm. A Luna 5 µ C18 (2) 100A (250 × 10.0 mm) semi preparative column with a C18 guard column (Phenomenex, Torrance, CA, USA) was used for the separation. The compound of interest was eluted using binary mobile phase at a flow rate of 1.0 mL/min with run time of 23 min. The mobile phase was acetonitrile:water (32:68 by volume) with 0.5% formic acid. A volume of 100 µL were injected. The retention time of the compound X ranged between 17 and 21 min. This elution fraction was collected using an Agilent 1200 series fraction collector. The collected fraction was concentrated by a rotary evaporator (Heidolph, Elk Grove Village, IL, USA) to remove solvent. Then it was freeze-died (Labconco, Kansas City, MO, USA) and the pure compound X was obtained.

### 5.8. Identification of ZEA Transformation Product by LC-MS/MS and NMR

LC/MS analysis of ZEA and compound X were carried out by using a Thermo Scientific Q-Exactive Benchtop Orbitrap Mass Spectrometer connected with a Vanquish™ Flex Binary UPLC System (Thermo Scientific, Waltham, MA, USA). An Agilent ZORBAX SB-C18 column (2.1 × 100 mm, 3.5 µm) was used for separation. Two mobile phases, solvent A (99.9% H_2_O + 0.1% formic acid) and solvent B (99.9% acetonitrile + 0.1% formic acid) were used. The chromatographic elution condition was as following: 0–10 min, isocratic 40% B. The column compartment was controlled at 25 °C, the flow rate was set at 0.4 mL/min, the injection amount was 10 µL and the UV absorption wavelength is 270 nm. The negative heated-electrospray ionization (HESI) mode was used for the data collection. The optimized HESI conditions were as follows: sheath gas, 58 arbitrary units; auxiliary gas, 16 arbitrary units; sweep gas, 3 arbitrary units; spray voltage, 3.5 kV; S-lens RF level, 50%; capillary temperature, 281 °C and auxiliary heater temperature, 463 °C. The normalized collision energy (NCE) used for parallel reaction monitoring (PRM) mode was set at 20%. The automatic gain control target and maximum injection time were 1e^6^ and 50 ms.

1D ^1^H and 2D spectra, such as Correlated Spectroscopy (COSY), Heteronuclear Single-Quantum Correlation (HSQC), Heteronuclear Multiple Bond Correlation (HMBC), and Rotating-frame Overhauser and Exchange Spectroscopy (ROESY), were acquired at 25 °C on an Agilent DD2 spectrometer (υ(^1^H) = 699.803 MHz, υ(^13^C) = 175.982; Agilent, Walnut Creek, CA, USA) equipped with a 5 mm HFCN cold probe. The 1D ^13^C spectrum was acquired at 25 °C on an Agilent DD2 spectrometer (υ (^1^H) = 499.662 MHz, υ (^13^C) = 125.653; Agilent, Walnut Creek, CA, USA) equipped with a 5 mm XSens cold probe. The 1D ^31^P and 2D Heteronuclear NOESY (HP-HOESY) spectra were acquired at 25 °C on an Agilent DD2 spectrometer (υ (^1^H) = 599.823 MHz, υ (^31^P) = 242.812 MHz; Agilent, Walnut Creek, CA, USA) equipped with a 5 mm HFX probe. Purified compound X (1.5 mg) was dissolved in a 1:1 mixture of acetonitrile-d3: D_2_O (550 µL total volume). The mixture was vortexed for 1 min and then transferred to a 5 mm tube for analysis by NMR. NMR spectra were processed using MestReNova (version 10.0.2-15465, Mestrelab Research S.L., Santiago de Compostela, Spain). 1H and 13C chemical shifts were referenced relative to acetonitrile- d3 (δ(^1^H) = 1.94 ppm, δ(^13^C) = 1.320 ppm).

### 5.9. Identification of ZEA Transformation Product by Comparing Standard ZEA-14-Phosphate and ZEA-16-Phosphate

Compound X was purified from the metabolites of *Bacillus* sp. S62-W using the above-mentioned method. Since neither compound of ZEA phosphate conjugation was reported, nor were such products available, ZEA-14-phosphate and ZEA-16-phosphate were artificially synthesized based on our hypothesized ZEA transformation products. In order to identify the position of phosphate conjugation, 50 µg/mL for each compound were analyzed using an HPLC system described above. The compound of interest was eluted using binary mobile phase at a flow rate of 1.0 mL/min. The mobile phase was acetonitrile:water with 0.5% formic acid (60:40 by volume) and the injection volume was 10 μL.

### 5.10. Determination of ZEA Phosphorylation in Various Bacillus Strains

Multiple *Bacillus* spp. were selected to test ZEA phosphorylation activities. Twenty-one strains (*B. amyloliquefaciens*, *B. licheniformis*, *B. megaterium*, *B. pumilus*, and *B. subtilis*; see Figure 8 for complete list of the strains) were obtained from the laboratory culture collection of the Guelph Research and Development Centre, Agriculture and Agri-Food Canada. Two strains, *B. pumilus* AQP-4275 and *B. subtilis* AQP-6638, were isolated by Lallemand Inc. (Montréal, QC, Canada), which are exempted of Nagoya protocol. The ZEA detoxification activity was identified using the techniques described above. Briefly, bacteria from −80 °C freezer were initially enriched in NA plates and were incubated for 3 days at 28 °C. The colonies were transferred into 1 mL of CMB and were incubated overnight at 28 °C with shaking at 120 RPM. In order to ensure the similar initial concentration of inoculum, the enriched cultures were normalized to the OD_600_ = 0.1. Then 50 µL of normalized culture was inoculated to 945 µL of fresh CMB and 5 µL of ZEA (5000 µg/mL). The final concentration of ZEA was 25 µg/mL. The control sample was prepared in the same method without inoculation of bacteria. Samples and control were incubated at 28 °C with shaking at 120 RPM. One hundred microliter of each sample was taken out after 24 h. The transformation was terminated by adding 600 µL of acetonitrile and 300 µL of water. All samples were filtered by 0.45 µm syringe filter before HPLC analysis.

### 5.11. Statistical Analysis

ZEA transformation activities of *Bacillus* sp. S62-W were performed in triplicate with completely independent and randomized design. The statistical analyses of experimental data were carried out by SPSS version 20 (IBM, Armonk, NY, USA). Statistical significance of ZEA concentration between control and treatment were evaluated through paired *T*-test. The percentages of ZEA transformation were evaluated through one-way ANOVA with post-hoc Tukey’s HSD test.

## Figures and Tables

**Table 1:** Putative identities of S62-W based on 16S rRNA gene sequence.

Microorganism	Accession No.	Identity (%)
*Bacillus aerius* 24K	NR118439.1	99
*Bacillus stratosphericus* 41KF2a	NR042336.1	99
*Bacillus altitudinis* 41KF2b	NR042337.1	99
*Bacillus pumilus* NBRC 12092	NR112637.1	99
*Bacillus pumilus* SAFR-032	NR074977.1	99
*Bacillus pumilus* ATCC 7061	NR043242.1	99
*Bacillus pumilus* SBMP2	NR118381.1	99
*Bacillus safensis* NBRC 100820	NR113945.1	99
*Bacillus safensis* FO-366	NR041794.1	99

**Table 2:** Putative identities of S62-W based on whole genome analysis with JSpeciesWS Tetra correlation search (TCS) tool.

Microorganism	Z-Scores	ANIb (%)
*Bacillus stratosphericus* LK31	0.99986	98.05
*Bacillus stratosphericus* (GCA_001043535) LK5	0.99984	98.07
*Bacillus stratosphericus* LK23	0.99982	98.06
*Bacillus* sp. LK10	0.99982	98.06
*Bacillus stratosphericus* (GCA_001038775) LK33	0.99981	98.06
*Bacillus* sp. L_1B0_12	0.99979	98.02
*Bacillus stratosphericus* (GCA_001038845) LK18	0.99979	98.05
*Bacillus* sp. TH007	0.99979	98.79
*Bacillus pumilus* (GCA_000828455) B4133	0.99975	98.30
*Bacillus stratosphericus* (GCA_001265125) M53	0.99975	98.09
*Bacillus cellulasensis* NIO-1130	0.99974	97.91
*Bacillus altitudinis* 41KF2b	0.99973	98.01
*Bacillus* sp. FJAT-21955	0.99971	98.51
*Bacillus pumilus* (GCA_000972685) W3	0.99965	98.10

ANIb: Average Nucleotide Identities.

**Table 3:** ^1^H, ^13^C chemical shift assignments and 2D correlations for ZEA-phosphate.

Atom	Chemical Shift (ppm)	COSY	HSQC	HMBC	ROESY
1C	171.27			3, 13, 15	
3C	74.47		3	5b, 4b, 5a, 19	
4C	35.22		4a, 4b	19, 6a, 6b, 5b, 5a, 3	
5C	22.31		5a, 5b	3, 19, 6a, 4b, 4a, 6b	
6C	43.85		6a, 6b	4b, 5b, 5a	
7C	216.18			6a, 6b, 8b, 8a, 9b, 5b, 9a, 5a	
8C	37.68		8b, 8a	6a, 9b, 9a, 6b, 10a, 10b	
9C	21.60		9b, 9a	11, 8b, 8a, 10a, 10b, 12	
10C	31.65		10b, 10a	11, 8a, 8b, 9b, 9a, 12	
11C	134.56		11	12, 9b, 9a, 10b, 10a	
12C	131.54		12	11, 13, 10b, 10a	
13C	112.04		13	12, 15	
14C	157.33			13, 15	
15C	107.35		15	13	
16C	161.26			15	
17C	110.12			15, 13, 12, 19	
18C	142.00			12, 11	
19C	20.41		19(H_3_)	4b, 3	
3H	5.01	19, 4b	3	5, 1, 4, 19	5b, 4a, 4b, 5a, 6a
4Ha	1.61	4b	4	5	3, 6a
4Hb	1.51	3, 4a, 5a	4	5, 6, 19, 3	12, 3, 6a, 6b
5Ha	1.62	5b, 4b, 6b, 6a	5	7, 4, 6, 3	3, 8a, 6a
5Hb	1.70	6a, 6b, 5a	5	7, 4, 6, 3	12, 3, 19, 8a, 8b, 6a
6Ha	2.45	6b, 5b, 5a	6	7, 8, 4, 5	3, 4b, 5b, 5a, 4a
6Hb	2.17	11, 6a, 5b, 5a	6	7, 4, 8, 5	8b, 4b
8Ha	2.38	8b, 9b, 9a	8	7, 9, 10	11, 9b, 5b, 9a, 5a
8Hb	2.58	8a, 9b, 9a	8	7, 9, 10	11, 5b, 6b, 9a
9Ha	1.63	9b, 8a, 8b, 10b, 10a	9	7, 10, 8, 11	11, 8a, 8b
9Hb	1.79	9a, 8b, 8a, 10b	9	7, 10, 8, 11	11, 8a, 10a
10Ha	2.11	11, 12, 10b, 9a	10	9, 8, 12, 11	12, 9b
10Hb	2.15	11, 12, 10a, 9b, 9a	10	9, 8, 12, 11	12
11H	5.89	12, 6b, 10a, 10b	11	9, 10, 18, 12	13, 9a, 9b, 8a, 8b
12H	6.71	11, 10a, 10b	12	18, 11, 13, 17, 10, 9	4b, 10a, 10b, 5b, 19
13H	6.75	15	13	12, 14, 1, 15, 17	11
15H	6.65	13	15	17, 14, 16, 1, 13	
19H_3_	1.29	3	19	4, 5, 3, 17	12, 5b

COSY: Correlated Spectroscopy; HSQC: Heteronuclear Single-Quantum Correlation; HMBC: Heteronuclear Multiple Bond Correlation; ROESY: Rotating-frame Overhauser and Exchange Spectroscopy.

## Data Availability

Not applicable.
